# Method for geological characteristics prediction during shield tunnelling: SCA-GS

**DOI:** 10.1016/j.mex.2022.101883

**Published:** 2022-10-20

**Authors:** Tao Yan

**Affiliations:** a MOE Key Laboratory of Intelligent Manufacturing Technology, Department of Civil and Environmental Engineering, College of Engineering, Shantou University, Shantou, Guangdong, 515063, China; b Discipline of Civil and Infrastructure, School of Engineering, Royal Melbourne Institute of Technology (RMIT), Victoria, 3001, Australia

**Keywords:** Stacking classification algorithm, Grid search, K-folds cross-validation, prediction

## Abstract

Geological characteristic (GC) is one of the most essential factors influencing setting earth pressure balance (EPB) shield parameters and cutterhead wear. Identification of GC has crucial significance to shield tunnelling efficiency and safety. Stacking classification algorithm (SCA) is widely applied in engineering with the identification and classification. Grid search (GS) is designed to tune hyper-parameter and optimize non-linear problems with K-folds cross-validation (K-CV), which is commonly used to change validation set in the training set. The performance of SCA can be improved by GS and K-CV. The types of GC during shield advance can be identified by integrating K-means++ with silhouette coefficient (*S_i_*) and elbow method (EM). The results of K-means++ and shield parameters severed as a database for SCA. The approach was applied in Guangzhou mixed ground. The results showed that the proposed framework could predict the geological characteristics well. The method article is a companion paper with the original article [1]. The proposed method enables:

• Developed approach merges SCA and GS method.

• Application of SCA-GS method in geological characteristics classification.

• It can increase the reliability of classification results.

## Graphical abstract

**Figure:**
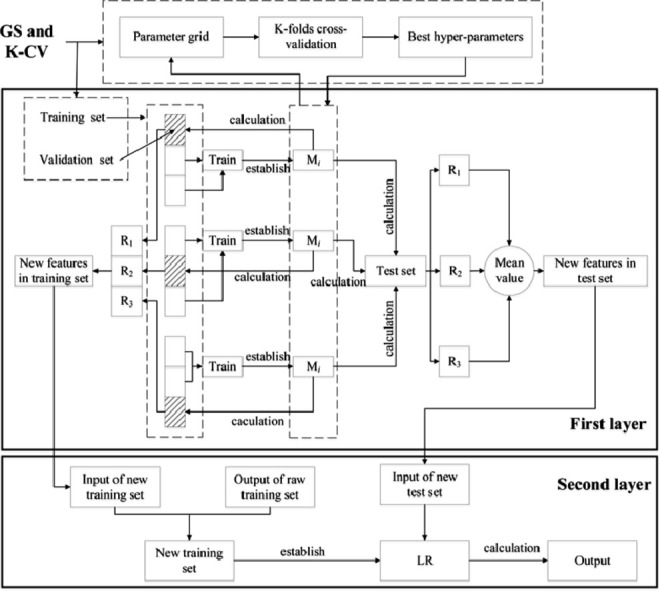

Specification Table

**Table:** 

Subject area:	Engineering Geology
More specific subject area:	Geological characteristics
Method:	Integrating SCA method with GS and K-CV
Name and reference of the original method:	Wolpert, D.H., (1992) Stacked generalization, Neural Networks, 5(2), 241-259, https://doi.org/10.1016/S0893-6080(05)80023-1 [2].
Resource availability:	DOI: https://doi.org/10.1016/j.jrmge.2022.03.002.

## Method details

### Stacking classification algorithm (SCA)

In the process of SCA, a variety of models were combined into an ensemble algorithm. The detail of SCA implementation is given [2]:

**Step 1:** Establish the database and pre-process data. The data with different characteristics are acquired, and the database matrix is constructed in Eq. (1).

(1) $$X={\left[x_{i,j}\right]}_{n\times m}=\left[\begin{array}{cccccc} & C_{1} & C_{2} & C_{3} & \cdots & C_{m}\\ X_{1} & x_{1,1} & x_{1,2} & x_{1,3} & \cdots & x_{1,m}\\ X_{2} & x_{2,1} & x_{2,2} & x_{2,3} & \cdots & x_{2,m}\\ X_{3} & x_{3,1} & x_{3,2} & x_{3,3} & \cdots & x_{3,m}\\ \vdots & \vdots & \vdots & \vdots & \vdots & \vdots \\ X_{n} & x_{n,1} & x_{n,2} & x_{n,3} & \cdots & x_{n,m} \end{array}\right]$$

where *C_m_* is the name of data *m; X_n_* denotes the *n^th^* sample in *C_m_*. Then, the mean value of 3 sequential points after time *t* is used to replace the value of time *t*. Meanwhile, the data in *X_n_* are normalized to the interval [0, 1] according to Min-Max Normalization. The normalized database is presented as follow:

(2) $$X^{\prime }={\left[{x^{\prime }}_{i,j}\right]}_{n\times m}=\left[\begin{array}{cccccc} & C_{1} & C_{2} & C_{3} & \cdots & C_{m}\\ {X^{\prime }}_{1} & {x^{\prime }}_{1,1} & {x^{\prime }}_{1,2} & {x^{\prime }}_{1,3} & \cdots & {x^{\prime }}_{1,m}\\ {X^{\prime }}_{2} & {x^{\prime }}_{2,1} & {x^{\prime }}_{2,2} & {x^{\prime }}_{2,3} & \cdots & {x^{\prime }}_{2,m}\\ {X^{\prime }}_{3} & {x^{\prime }}_{3,1} & {x^{\prime }}_{3,2} & {x^{\prime }}_{3,3} & \cdots & {x^{\prime }}_{3,m}\\ \vdots & \vdots & \vdots & \vdots & \vdots & \vdots \\ {X^{\prime }}_{n} & {x^{\prime }}_{n,1} & {x^{\prime }}_{n,2} & {x^{\prime }}_{n,3} & \cdots & {x^{\prime }}_{n,m} \end{array}\right]$$

where *X′* and *x′* are the normalized value corresponding to Eq.(1).

**Step 2:** Determine types for the database. The database types can be labelled by K-means++ algorithm [3]. Euclidean distance is applied in K-means++, which can be expressed as Eq. (3).

(3) $$dist\left(A,B\right)=\sqrt{\sum_{i=1}^{n}|a_{i}-{b_{i}|}^{2}}$$

where *dist*(*A, B*) represents the distance of samples A and B; *a_i_* and *b_i_* denote the coordinates of samples A and B. The square error is utilized as the objective function in K-means++. The objective function is a convex function of cluster centers (*μ*_1,_ *μ*_2_,…, *μ_k_*) and the stagnation point of the objective function is the clustering centers. The objective function and cluster centers can be expressed as follows [4]:

(4) $$J\left(\mu_{1},\mu_{2},...,\mu_{k}\right)=\frac{1}{2}\sum_{j=1}^{K}\sum_{i=1}^{N_{j}}{\left(x_{i}-\mu_{j}\right)}^{2}$$

(5) $$\frac{\partial J}{\partial \mu_{j}}=-2\sum_{i=1}^{N_{j}}\left(x_{j}-\mu_{j}\right)\to 0\Rightarrow \mu_{j}=\frac{1}{N_{j}}\sum_{i=1}^{N_{j}}x_{j}$$

where *J*() is the objective function; *μ_j_* is the center of cluster *j; N_j_* denotes the sample number of category *j; x_i_* represents a random sample in the database; *x_j_* is the sample point of cluster *j*. The elbow method (EM) and silhouette coefficient (*S_i_*) are utilized to find the value of K in K-means++. The diagram of the sum of the squared errors (*SSE*) versus K in elbow method will form an elbow, and the corresponding K to the elbow of the diagram is determined as the optimal cluster number. When there are more than one elbow in elbow method, K in elbow method corresponding to the maximum silhouette coefficient (*S_i_*) can be selected as the best value for the K-means++. The *SSE* and silhouette coefficient (*S_i_*) can be calculated by Eq. (4) and Eq. (5) in the companion paper [1]. The value of K types can be set as 1to K corresponding to different values of *X_n_*. Then, each *X_n_* will be labelled with the value of K ($L_{k}^{n}$) in the matrix in Eq. (6).

(6) $$X_{k}^{\prime }={\left[{x^{\prime }}_{i,j},L\right]}_{n\times (m+1)}=\left[\begin{array}{cccccc} & C_{1} & C_{2} & C_{3} & \cdots & C_{m}\\ {X^{\prime }}_{1} & {x^{\prime }}_{1,1} & {x^{\prime }}_{1,2} & {x^{\prime }}_{1,3} & \cdots & {x^{\prime }}_{1,m}\\ {X^{\prime }}_{2} & {x^{\prime }}_{2,1} & {x^{\prime }}_{2,2} & {x^{\prime }}_{2,3} & \cdots & {x^{\prime }}_{2,m}\\ {X^{\prime }}_{3} & {x^{\prime }}_{3,1} & {x^{\prime }}_{3,2} & {x^{\prime }}_{3,3} & \cdots & {x^{\prime }}_{3,m}\\ \vdots & \vdots & \vdots & \vdots & \vdots & \vdots \\ {X^{\prime }}_{n} & {x^{\prime }}_{n,1} & {x^{\prime }}_{n,2} & {x^{\prime }}_{n,3} & \cdots & {x^{\prime }}_{n,m} \end{array}\begin{array}{c} Label\\ L_{k}^{1}\\ L_{k}^{2}\\ L_{k}^{3}\\ \vdots \\ L_{k}^{n} \end{array}\right]$$

**Step 3:** Input primary learners, meta-classifier and establish stacking classification model. SCA includes two layers, primary learners and meta-classifier. Support vector machine (SVM), random forest (RF), and gradient boosting decision tree (GBDT) are utilized in the primary learners [5]. The logistic regression algorithm (LR) is employed as a meta-classifier. The results of the first layer with more characteristics can be taken as the input in the second layer to obtain higher accuracy for the proposed model. The results of the first layer can be constructed in Eq. (7).

(7) $$X_{k,3}^{\prime }={\left[{x^{\prime }}_{i,j},L\right]}_{n\times (m+3)}=\left[\begin{array}{cccccc} & C_{1} & C_{2} & C_{3} & \cdots & C_{m}\\ {X^{\prime }}_{1} & {x^{\prime }}_{1,1} & {x^{\prime }}_{1,2} & {x^{\prime }}_{1,3} & \cdots & {x^{\prime }}_{1,m}\\ {X^{\prime }}_{2} & {x^{\prime }}_{2,1} & {x^{\prime }}_{2,2} & {x^{\prime }}_{2,3} & \cdots & {x^{\prime }}_{2,m}\\ {X^{\prime }}_{3} & {x^{\prime }}_{3,1} & {x^{\prime }}_{3,2} & {x^{\prime }}_{3,3} & \cdots & {x^{\prime }}_{3,m}\\ \vdots & \vdots & \vdots & \vdots & \vdots & \vdots \\ {X^{\prime }}_{n} & {x^{\prime }}_{n,1} & {x^{\prime }}_{n,2} & {x^{\prime }}_{n,3} & \cdots & {x^{\prime }}_{n,m} \end{array}\begin{array}{c} Lable_{1}\\ L_{k,1}^{1}\\ L_{k,1}^{2}\\ L_{k,1}^{3}\\ \vdots \\ L_{k,1}^{n} \end{array}\begin{array}{c} Label_{2}\\ L_{k,2}^{1}\\ L_{k,2}^{2}\\ L_{k,2}^{3}\\ \vdots \\ L_{k,2}^{n} \end{array}\begin{array}{c} Label_{3}\\ L_{k,3}^{1}\\ L_{k,3}^{2}\\ L_{k,3}^{3}\\ \vdots \\ L_{k,3}^{n} \end{array}\right]$$

where $L_{k,m}^{n}$ is the label of sample *n* in model *m* (*m* = 1, 2, 3). The radial basis function (rbf) in SVM, the method of weighted mean in RF, the multi-classification loss function in GBDT are expressed as Eq. (8)-(10) and applied to classify the multiple geological characteristics [6], [7], [8].

(8) $$\kappa \left(x_{i},x_{j}\right)=\exp\left(-\frac{{∥x_{i}-x_{j}∥}^{2}}{2\sigma^{2}}\right)$$

(9) $$G\left(x\right)=\sum_{i=1}^{m}w_{i}g_{i}\left(x\right)$$

(10) $$L\left(y,f_{t}\left(x\right)\right)=-\sum_{k=1}^{K}y_{k}\log p_{k}\left(x\right),y_{k}\in \left(-1,1\right)$$

where *κ*() is the kernel function; *x_i_, x_j_* are sample points; *G*(*x*) is the output according to weight of *x; w_i_* is the weight of decision trees; *g_i_*(*x*) is the value of decision trees; *L*() represents the loss function; *f_t_*(*x*) is the output function; *y* denotes the label of point *x; p_k_*(*x*) represents the probability for sample *x* in class *k*.

**Fig. 1:**
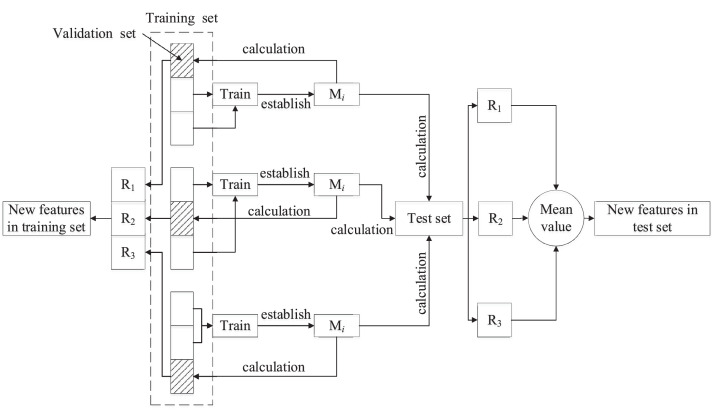
First layer running process in stacking classification algorithm (SCA).

**Fig. 2:**
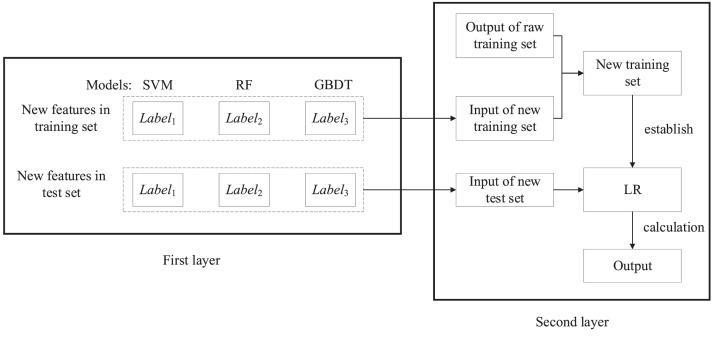
Second layer running process in stacking classification algorithm (SCA).

**Step 4:** Train the stacking classification model and predict. The classification model will be trained and evaluated using cross-validation and training set. Fig. 1 indicates the frame of the first layer running process in stacking algorithm (taking 3-CV as a case). The training set is input into the first layer to train the primary learners and establish the primary models (M*_i_*). The new features in training set and test set will be calculated via the primary models using the validation set in raw training and test sets. Fig. 2 shows the second layer running process in stacking classification algorithm. The new features in training set are taken as input of new training set. The new training set is composed of input of new training set and output of raw training set, which are applied to train the classifier in second layer (LR) and establish a prediction model. Then, the new features in test set are taken as input of new test set and are input into the prediction model to output the final results.

**Step 5:**Evaluate the stacking classification algorithm model. The output GC of the SCA algorithm will be compared with the actual geological characteristics of raw test set. The F1-score, recall (*R*), and precision (*P*) are used to assess the proposed strategy.

### Grid search with cross-validation

**Fig. 3:**
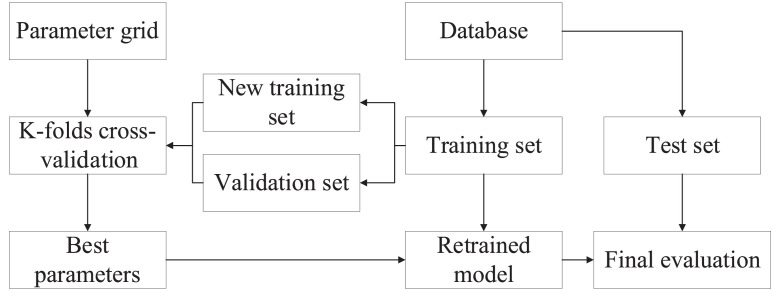
Flowchart of grid search and K-folds cross-validation.

Grid search is an exhaustive search method of selecting parameters. In all candidate parameters, every possibility will be tried, and the best performance of parameter combination will be selected as final parameters of the model. However, the database is divided into test and training set, and the model with the default hyper-parameters are trained using training set, which has low accuracy. The grid search combined with cross-validation can solve the problem of high error for the model. The database is divided into three parts: test, training, and validation set. The proposed framework can be fitted by training set and verified on validation set. Test set is applied in assessing the model. To avoid the influence of database division, cross-validation is employed to reduce contingency of database division. K-folds cross-validation is commonly used to change validation set in the training set. Grid search is usually applied together with K-CV. Fig. 3 shows the flowchart of GS and K-CV.

### Application of the method

The SCA-GS model can be used to classify the GC during shield advance. Four shield parameters (cutterhead rotation speed (CRS), advance rate (AR), shield thrust (F), and cutterhead torque (T)) were collected from the sensors installed in EPB shield machine [9], [10], [11]. The empty data is removed according to Eq. (11) and Eq. (12) [12].

(11) D=f(F)·f(AD)·f(CRS)·f(T)

(12) $$f\left(x\right)=\left\{ \begin{array}{c} 0,x=0\\ 1,x\neq 0 \end{array}\right.$$

where if *D* = 1, data was saved. If *D* = 0, data was excluded. *x* is the value of shield parameters. The abnormal value out of three times standard deviation from the mean value was removed with Pauta criterion. The original data matrix is constructed in Eq. (13).

(13) X=[xi,j]n×4=[FADCRSTX110.5211.12.5X227.5151.25.7X3⋮27⋮10⋮1.8⋮3⋮X117918.7351.32.3]

where *F* is shield thrust (× 10^3^ kN); *AR* is advance rate (mm/min); *CRS* is cutterhead rotation speed (rpm); *T* denotes cutterhead torque (× 10^3^ kN·m). Then, the raw data were transformed into torque penetration index (TPI) and field penetration index (FPI) [13, 14], which can be obtained by Eq. (6) in the associated paper [1]. The mean value of 3 sequential points after time *t* replaced the value of time *t*. Meanwhile, TPI and FPI are normalized to the interval [0, 1] according to Min-Max Normalization. The normalized matrix is taken as input data and constructed in Eq. (14).

(14) $$X^{\prime }={\left[{x^{\prime }}_{i,j}\right]}_{n\times 2}=\left[\begin{array}{ccc} & FPI & TPI\\ {X^{\prime }}_{1} & 0.279 & 0.014\\ {X^{\prime }}_{2} & 0.120 & 0.142\\ {X^{\prime }}_{3} & 0.289 & 0.172\\ \vdots & \vdots & \vdots \\ {X^{\prime }}_{1179} & 0.023 & 0.012 \end{array}\right]$$

Geological characteristics are labelled by K-means++ algorithm with elbow method (EM) and silhouette coefficient (*S_i_*) using normalized FPI and TPI. Then, according to the site investigation of the construction site, the GCs in the project can be identified as K types, and the value of K types can be set as 1 to K, corresponding to different values of FPI and TPI, respectively. The geological characteristics in each tunnelling ring were labelled with the value of K ($L_{n}^{k}$) in Fig. 8 in the companion paper [1], and the database matrix is constructed in Eq. (15).

(15) $$X_{k}^{\prime }={\left[{x^{\prime }}_{i,j},L\right]}_{n\times 3}=\left[\begin{array}{ccc} & FPI & TPI\\ {X^{\prime }}_{1} & 0.279 & 0.014\\ {X^{\prime }}_{2} & 0.120 & 0.142\\ {X^{\prime }}_{3} & 0.289 & 0.172\\ \vdots & \vdots & \vdots \\ {X^{\prime }}_{1179} & 0.023 & 0.012 \end{array}\begin{array}{c} Label\\ 1\\ 2\\ 3\\ \vdots \\ 1 \end{array}\right]$$

The database in Eq. (15) was split into training set with 80% and test set with 20% of data set and were input to the SCA-GS model. The optimal value of parameters and accuracy of primary learners are given in Table 3 in the companion paper [1]. The algorithms in the first layer with optimal hyper-parameters are trained and tested using training and test sets. The results of the first layer can be constructed in Eq. (16).

(16) $$X_{k3}^{\prime }={\left[{x^{\prime }}_{i,j},L\right]}_{n\times 5}=\left[\begin{array}{ccc} & FPI & TPI\\ {X^{\prime }}_{1} & 0.279 & 0.014\\ {X^{\prime }}_{2} & 0.120 & 0.142\\ {X^{\prime }}_{3} & 0.289 & 0.172\\ \vdots & \vdots & \vdots \\ {X^{\prime }}_{1179} & 0.023 & 0.012 \end{array}\begin{array}{c} Lable_{1}\\ 1\\ 2\\ 3\\ \vdots \\ 1 \end{array}\begin{array}{c} Label_{2}\\ 1\\ 2\\ 3\\ \vdots \\ 1 \end{array}\begin{array}{c} Label_{3}\\ 1\\ 2\\ 3\\ \vdots \\ 1 \end{array}\right]$$

The final output of prediction model was given in Fig. 13 in the companion paper [1]. The performance of SCA-GS prediction model was improved with the highest accuracy (0.996), which satisfies the requirement of shield tunnelling. Therefore, the prediction model can be utilized in a new project.

## Computational tool

**Fig. 4:**
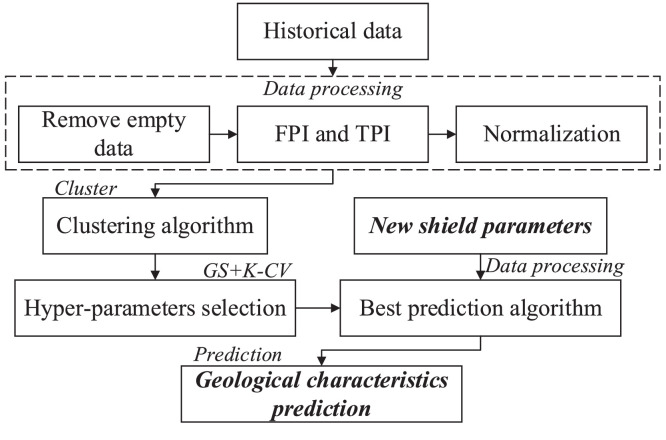
Flowchart of geological characteristics prediction.

The study used Python program to establish the SCA-GS prediction model for geological characteristics during shield tunnelling. The pseudocode of improved stacking classification algorithm is listed in the Appendix. The source code of the SCA-GS includes cluster, optimization and prediction modules. Fig. 4 shows the flowchart of geological characteristics prediction. The detailed steps of the method application are presented as follows:

(1) The users should prepare the dataset, which consists of the historical shield parameters (F, AR, CRS, and T) collected from shield operational system. Then, the empty data were removed based on Eq. (11) and (12).
(2) The shield parameters were calculated as FPI and TPI, which were normalized to the interval [0, 1] and input into the first part of the source code (cluster module). The users can change the data path of source code to input them.
(3) The users can select the first cluster module to run the K-means ++ algorithm to give each line a label (GC). The results of the cluster module were saved as the data set for the following steps.
(4) The data path was changed as the path of the results of K-means ++. The data set was input into the optimization module, which was used for training and testing prediction algorithms. The optimizer (GS and K-CV) will optimize the primary learners and provide the best hyper-parameters for prediction module.
(5) The hyper-parameters of the prediction module were replaced based on the results of step (4). As such, the best prediction model was established with the highest accuracy of prediction algorithms.
(6) The new shield parameters can be obtained during shield advance. Then, the users can set the input data path as the processed shield parameters and run the best prediction model to forecast real-time geological characteristics

Readers can contact the author to apply for the source code.

## Method validation

To verify the advantage of the designed SCA-GS method, the existing SVM, RF, GBDT, and SCA-GS techniques were used to recognize the relationship between GC and shield parameters in Fig. 13 in the companion paper [1]. Based on the accuracy and performance of four classification algorithms, the SCA-GS is the better prediction model for geological characteristics. The detailed comparison and analyses of prediction results can be found in the companioned research article [1].

Identifying geological characteristics is crucial for shield tunnelling and reducing construction risk [15], [16], [17]. However, there is no globally accepted mechanical relationship between shield parameters and geological characteristics. The variation of shield parameters is a gray-box process based on geological features. Many factors, e.g., ground settlement, underground water, and lining quality, may influence the tunnelling process [18], [19], [20]. Artificial intelligence, including expert systems, machine learning, and deep learning, is an excellent technique for establishing the relationship between various parameters and objectives [21], [22], [23], [24]. Four shield parameters were selected in this study to classify GC during shield advance. Besides, more parameters, e.g., specific energy, cutter wear, and earth pressure, should also be considered to evaluate their contribution to the prediction of GC [25].

## CRediT authorship contribution statement

**Tao Yan:** Data curation, Methodology, Investigation, Software, Writing – original draft, Writing – review & editing.

## Declaration of Competing Interest

The authors declare that they have no known competing financial interests or personal relationships that could have appeared to influence the work reported in this paper.

## Figures and Tables

**Table A1:** Pseudocode of SCA-GS.

**Algorithm** SCA-GS classification algorithm
**1: input:** training data *A* = {(*a*_1_, *b*_1_), (*a*_2_, *b*_2_),…, (*a_m_, b_m_*)};
primary learner: Μ_1_, Μ_2_, …, Μ*_S_*;
meta-classifier: Μ.
2. **for***s*= 1, 2, …, *S***do**
Param*_s_*={*p*_1_: (*a*_1_, *b*_1_, *t*_1_), *p*_2_: (*a*_2_, *b*_2_, *t*_2_), …*p*_n_: (*a*_n_, *b*_n_, *t*_n_)}
GS*_s_*=grid search (M*_s,_* Param*_s_*, K-CV)
**3:for***s*= 1, 2, …, *S***do**
**for***j* = 1, 2, …, *m***do**
M*_s_*′=fit(GS*_s_*(*a_j_*))
M*_s_*′′=high_accuracy(M*_s_*′)
**end for**
**end for**
**4. for***s*= 1, 2, …, *S***do**
*r_s_*= M*_s_*′′(*A*);
*A*′ = ∅;
**end for**
**5. for***j* = 1, 2, …, *m***do**
**for***s*= 1, 2, …, *S***do**
*u_js_* = *r_s_*(*a_j_*);
**end for**
*A*′ = *A*′∪((*u_j_*_1_, *u_j_*_2_,…,*u_jS_*,), *b_j_*)
**end for**
**6.***r*′ = M(*A'*)
**7. Output:***R*(*a*) = *r*'(*r*_1_(*a*), *r*_2_(*a*),…, *r_S_*(*a*))

## Appendix

Table A1.
